# A survey of dairy cattle farmers' management practices for cull cows in Ontario, Canada

**DOI:** 10.3389/fvets.2022.974061

**Published:** 2022-08-30

**Authors:** Joanne Marshall, Derek Haley, Lena Levison, David F. Kelton, Cynthia Miltenburg, Steven Roche, Todd F. Duffield

**Affiliations:** ^1^Department of Population Medicine, University of Guelph, Guelph, ON, Canada; ^2^Campbell Centre for the Study of Animal Welfare, University of Guelph, Guelph, ON, Canada; ^3^Dairy at Guelph, University of Guelph, Guelph, ON, Canada; ^4^ACER Consulting Limited, Guelph, ON, Canada; ^5^Ontario Ministry of Agriculture, Food and Rural Affairs, Elora, ON, Canada

**Keywords:** culling, compromised cow, cattle transportation, cull dairy cow, farm management

## Abstract

Removal of cows from dairy cattle production is a routine and unavoidable practice of the dairy industry and is often referred to as culling. The objectives of this study were to use a survey to describe current on-farm cull cow management, farmers' perception of cull cows' journeys to slaughter, and the adoption of current recommendations and regulations by Ontario dairy farmers. All Ontario dairy farmers were invited to complete a cull cow management survey between December 2020 and March 2021 that included 44 questions covering farmer demographic information, farm characteristics, and cull cow management. The survey response rate was 7.4% (*n* = 248); a total of 226 of the responses were included in this study for analysis. Most respondents indicated they have a written standard operating procedure (SOP) for cull cows (62%), and 48, 13, and 15% of those identified they use their cull cow SOP “always,” “sometimes,” and “never,” respectively. The more confident respondents were that cull cows arrived at slaughter in the condition they left the farm the less likely they were to have a cull cow SOP [odds ratio (OR) 0.83]. The most important sources of information for the management of cull cows were the herd veterinarian (64%) and members of the marketing/regulatory organization the Dairy Farmers of Ontario (44%). Drug withdrawal time was the only factor most respondents (73%) considered “very important” for the assessment of cull cows prior to transport. Most farmers believe cull cows journey from the farm to slaughter is three or less days (55%), and the confidence of farmers that cull cows arrive at slaughter in the condition they left their farm was generally high. Lastly, most farmers (66%) identified they were familiar with recent regulatory changes around the fitness, duration of transport, and lactation status for cull cows. These results highlight farmers' perceptions of the impacts and durations of the journey of cull cows differs from reality, and there are misunderstandings of the requirements for cull cow management. Further research should investigate how different strategies for training farmers may lead to improved cull cow welfare and regulatory compliance.

## Introduction

In dairy cattle production, removal of animals from the herd is a routine practice often referred to as culling. Culling is defined as the removal of a cow from the herd with the intent for sale, slaughter, salvage, or death (1). In Canada, the process of managing cull cows that will be leaving a farm with the intent for slaughter requires the use of standard operating procedures (2, 3). Dairy farmers often have an ever-changing list of cull candidates under observation for health or performance concerns called a cull list. Cows may be otherwise healthy but culled for poor reproductive performance or low production, while others were culled due to injury or clinical disease (4–6). In North America, the rate of culling on dairy farms is about 30% of the milking herd annually (5, 7). Despite the perceived benefits of extending the productive life of a dairy cow (production of milk and replacement heifers), there have been global trends toward lower cow longevity in dairy herds (8, 9). Oftentimes, decreased cow longevity has been pointed to as being due to the availability of replacement heifers, which has resulted from improved reproductive technologies and farm goals being achieved by genetic improvement (1, 9–11). At 15.2%, poor reproduction was the most common reported reason for the removal of cows from a herd (5, 7). However, cows culled for the reason of poor reproductive performance often have underlying diseases, making dairy cattle health is the primary reason for culling (12). Other commonly given primary reasons for culling include mastitis (9.0%), feet and leg problems (6.1%), and low milk production (7.5%) (5). These conditions pose risks to cow welfare by lowering the ability of a cow to withstand the challenges of the journey from the farm to slaughter (13, 14)].

The transportation of cull cows may require shipment across long distances, interactions with unfamiliar animals, deprivations of feed and water, engorged udders due to prolonged milking intervals, handling by various people, exposure to non-preferable temperatures, and multiple novel environments (6, 14, 15). These transportation factors lead to a risk of an increased severity of disease conditions, and therefore, decisions about when and where to ship any individual cull cow will be important to animal welfare.

Several studies have reported a high prevalence of disease conditions among cattle at auction yards in North America (13, 16–18). As reported by Moorman et al. (18), culled cows were found with unacceptable hock injuries, body conditions, and gaits 27, 41, and 73% of the time, respectively. Addressing this issue starts with the dairy farmer because the decision to ship a cull cow begins at the dairy farm (6). Financial incentive is often pointed to as the primary reason for the shipment of unfit cattle by farmers and buyers (6). Although strongly influential to management decisions, the potential financial outcomes of culling cows are not the only factors considered by farmers. Farmers must also consider factors like their past experiences, individual animal's histories, welfare outcomes, and regulatory requirements. Therefore, culling decisions are complex, and they required educated management decisions for some cattle, which may explain why some cattle with poor fitness are transported off farm (19, 20).

National codes of practice (e.g., Code of Practice for the Care and Handling of Dairy Cattle), quality assurance programs, and regulations have been made to try to address the issue of cull cow welfare (2, 21, 22). Updates to Canadian regulations for the inclusion of animals destined for slaughter in welfare checks during transport and milking of lactating animals at specific intervals, reflect legislation in Europe for the transportation of animals (22, 23). Yet in both regions, research suggests there is a low likelihood of financial penalty for shipment of unfit or compromised cows (18, 24). The frequency of unacceptable health scores of cull cows at livestock auctions and condemnation of carcasses post slaughter, indicates insufficient enforcement of existing rules, a lack of disincentives to refrain from the shipment of unfit animals, and a gap in knowledge for bovine veterinarians and farmers (12, 18). The current study objective was to use a survey to describe current on-farm cull cow management, farmers' perception of cull cows' journeys to slaughter, as well as the adoption of current recommendations and regulations by Ontario dairy farmers.

## Methods

### Study design

This project was conducted in collaboration with several researchers at the Ontario Veterinary College to complete investigation into four different research topics (cull cows, down cows, calves, antimicrobial use). Human Ethics approval was granted from the University of Guelph (Guelph, ON, Canada; REB no. 20-09-001).

This was an observational, cross-sectional study conducted using a survey to collect information. The provincial survey was available to all Ontario dairy farmers between December 2020 and March 2021 for data collection. The survey was developed by dairy researchers to address previously developed questions on key management practices. The survey was reviewed by 10 members of the research group and pretested by seven farmers. The final survey was available in English and consisted of 162 questions, which were divided into the following sections: farmer background information, farm information, cull cows, down cows, calf management, disease control and surveillance, antimicrobial use, and social values (Supplemental File S1; https://doi.org/10.5683/SP3/RC5HQZ). This paper will describe the outcomes of the farmer background information, farm information, and cull cows, which includes 44 questions from the survey.

Recruitment was completed in collaboration with the Dairy Farmers of Ontario (**DFO**) through magazine inserts and an advertisement for their members on their website. All Ontario dairy farmers are members of the DFO as it is required for their license and payment. A $10 gift card was offered as an incentive to complete the survey and was available to the first 250 responses. The survey was available to be completed online (Qualtrics; https://www.qualtrics.com/) or by telephone. During phone surveys, the survey administrator introduced themselves, explained the informed consent process, asked the survey questions as they were written with repeating when asked, and the responses were recorded directly into Qualtrics as a survey response; these responses were not distinguished in any way from the online submissions for analysis. Telephone interviews were entered manually into Qualtrics. A target response of approximately 345 surveys was initially set based on there being approximately 3,300 dairy producers in the province, a confidence of 95% and an allowable error for dichotomous questions of 5%.

### Statistical analyses

Data from the survey were downloaded from the survey software, imported, and cleaned in a Microsoft Excel (Microsoft Corporation 2018, Redmond, WA) spreadsheet. Cleaning of data consisted of renaming and labeling variables, transforming of variable like Linkert scales, and converting variables from text to numeric values to allow for the dataset to be best suited for immediate analysis within the data analysis software program. The cleaned data was then imported into STATA (STATA/IC version 16, StataCorp) for analysis. A data analysis plan (Supplemental File S2; https://doi.org/10.5683/SP3/RC5HQZ) was created for all analyses, including strategies for regression model building and thematic analysis for the open text responses.

Descriptive analyses were performed on all quantitative variables. The primary variables of interest included the self-reported number of cattle culled for primary reasons on the farm (cow temperament, current cull cow price, genetic potential, lameness, other disease/injury/illness, mastitis or somatic cell count, quota management, reproductive status, and milk farmer), beliefs of cull cow travel duration (same day, up to 7 days, 8 to 14 days, 15 to 21 days, and more than 21 days) and destinations (sales barn, slaughter plant, location determined by transporter, unsure, and other), the management of these cattle before leaving the farm (special management practices and contributing factors to date of culling), cull cow decision makers (owner, manager, family member other than owner, employee, veterinarian, and other), cull cow transporters (herd owner, manager, herd worker, hired transporter, and other), knowledge of transport regulations and satisfaction with that knowledge, the importance of difference factors to obtaining information on cull cow management (blogs/online forums/LISTERVS, extension personnel from the Ontario Ministry of Agriculture, Food and Rural Affairs (**OMAFRA**), Lactanet DHI, DFO, other farmer organizations, magazines/newsletters, scientific journals, social media, researchers, nutritionists or feed suppliers, veterinarians, websites/search engines, and other), self-reported confidence of cull cow conditions during the journey to slaughter (scale of 1 to 10; with 10 being very confident), and the existence, development, and use of cull cow standard operating procedures (**SOP**) along with the included components (body condition score, temperature, lameness, lactation status, drug withdrawal, mastitis, reproductive status, other illness or injury, and ability to stand). All questions using a 5-point Likert scale were collapsed into three categories: “very important/important,” “moderately important,” and “of little importance/unimportant” (25, 26) to simplify analyses and presentation.

With the use of logistic regression, the relationship between explanatory variables and the outcomes familiarity with transport regulations, having a cull cow SOP, and having had a refusal for transport were investigated. For continuous variables, the assumption of linearity was assessed, and if the linearity assumption was not met, the variable was categorized. To investigate collinearity among the explanatory variables, Spearman rank coefficients were generated. If the correlation coefficient between two variables was beyond the range of ≥0.7 to ≤−0.7, the more biologically plausible variable was retained. Univariate analyses were performed for each model with a liberal *P*-value (<0.20) for a cutoff to screen for predictor variables with unconditional associations with the outcome. We also considered the causal models, scientific plausibility, and the meaningfulness of the association. Once these were determined to meet statistical and scientific merit the predictors were subsequently offered to multivariable models. Independent variables of potential significance from univariate analyses were offered to the multivariable model with consideration and inclusion of confounders. The final model was constructed using manual backward stepwise regression and included significant variables at a *P* ≤ 0.05. In repeated measures models, pairwise comparisons were used to evaluate predictive margins. Following construction of a reduced multivariable regression, variables with suspected interaction, based on the researcher's knowledge, were evaluated for two-way interactions of significance. Confounders were identified through construction of causal diagrams, and as variables that when removed from the model, changed the coefficients of the remaining variables by more than 20%. Visual assessment of outliers was done using scatter plots of standardized residuals, and the Hosmer-Lemeshow goodness-of-fit test was used to evaluate model fit.

To assess how information sources may impact management decisions made by farmers when culling cows, the self-reported assigned importance of sources for obtaining information about cull cow management was compared with the self-reported level of importance assigned to factors for assessing cow fitness for transport immediately before loading using a Kruskal-Wallis equality-of-populations rank test, with a *P*-value of <0.01 considered to be significant. Following these pairwise comparisons, Dunn's pairwise tests with Bonferroni adjustment correction were carried out *post-hoc* (27).

## Results

A total of 248 survey responses were collected. This represents a response rate from the entire Ontario dairy industry of 7.4% (248/3,340). Most respondents completed the survey online (98.8%), and three respondents (1.2%) completed the survey by phone. A total of 22 survey responses were removed due to non-consent for participation (seven respondents), non-eligibility (six respondents self-identified as not currently dairy farming in Ontario) and dropping out after consent and not identifying as a current Ontario dairy farmer (nine respondents). The remaining 226 respondents were included in most analyses. For cull cow related questions, two respondents were removed due to their self-reporting of not being involved in cull cow management, leaving 224 responses for analyses. Standard operating procedure-related questions had 182 responses included for descriptive analyses since 44 respondents indicated their farm did not have a cull cow SOP. Respondents were permitted to skip any questions, so frequency counts were not equal for all questions. Personal and farm demographic characteristics of the study population are presented in Table 1.

**Table 1:** Comparison of survey respondents in the study to the overall Ontario, Canada dairy industry (complied from a variety of sources) by personnel and farm-level demographic characteristics.

**Item**	**Study population (*n* = 226), no. (%)**	**Ontario, Canada dairy industry (*n* = 3,367^a^), no. (%)**
Age (yr)^b^^*^		
<30	45 (19.9)	340 (8.6)
30–39	65 (28.8)	621 (15.7)
40–49	47 (20.8)	1,182 (29.9)
50–59	47 (20.8)	1,115 (28.2)
≥60	22 (9.7)	696 (17.6)
Gender^c^^**^		
He/him	154 (68.1)	5,740 (63.8)
She/her	68 (30.1)	3,250 (36.2)
They/them	3 (1.3)	
**Education^c^**		
Elementary school	10 (4.4)	1,240 (22.0)
Secondary school	46 (20.4)	1,690 (30.0)
Apprenticeship, professional degree	6 (2.5)	250 (4.4)
College, CEGEP	90 (39.7)	1,785 (31.7)
University, postgraduate degree	75 (33.0)	660 (11.7)
**Organic status^d^**		
Yes	7 (3.0)	82 (2.6)
No	199 (88.1)	3,285 (97.4)
**Lactating herd size^e^**		
Mean	123.0	78.0
SD	132.0	
**305 d milk yield^f^**		
Mean	10, 729.6	12, 582
SD	2,003.7	
Breed^***^^g^		
Holstein	203 (89.8)	(94.6)
Jersey	35 (15.5)	(4.2)
Other	29 (12.8)	(2.2)
**Housing^h^**		
Tiestall	57 (25.2)	1,314 (67.2)
Freestall	141 (62.4)	642 (32.8)
Pack	8 (3.5)	
**Milking system^i^**		
Pipeline	59 (26.1)	1,314 (67.2)
Parlor	93 (41.2)	495 (25.3)
Robotic milking system	53 (23.5)	147 (7.5)

a Canadian Dairy Information Center, Government of Canada, 2020.

b Demographic changes in Canadian Agriculture, Statistics Canada, 2011.

* Age no. calculated using national values multiplied by proportion of dairy farms of Canada located in Ontario.

c Data tables, 2016 Census. Statistics Canada.

** Statistics Canada reports only binary sex - not gender identification.

d Socioeconomic overview of the farm population. Dairy cattle and milk production. Ontario. Statistics Canada. 2018.

e Canadian Dairy Information Center, Government of Canada.

f Lactanet, 2021.

g Ministry of Agriculture, Food and Rural Affairs.

*** Breeds Given by number of farms with each breed within their herd. Each farm may be composed of more than one breed of cattle.

h Canadian Dairy Information Centre, Government of Canada, 2020.

i Canadian Dairy Information Centre, Government of Canada, 2020.

### Culling decisions

Most culling decisions for dairy herds in Ontario were made by owners (69.2%; *n* = 176). The average proportion of cows culled identified by respondents ranged between 7.3 and 33.1%. Some respondents (7.1%; *n* = 12) identified milk production, reproductive status, quota management, or current cow price as being a singular primary reason for culling more than 75% of the cull cows from their herd. Between 46.2 to 98.9% of respondents identified each singular reason for culling as being responsible for <25% of the culling decisions made for their herd. Most respondents identified drug withdrawal times (52.2%; *n* = 117) and the reason a cow is being culled (61.2%; *n* = 137) as contributing factors to deciding when a cull cow leaves the farm. Fewer respondents identified the availability of transportation (37.1%; *n* = 83) and the day of the week (48.2%; *n* = 108) as being contributing factors to deciding when a cull cow leaves the farm.

### Cull cow management

Only two respondents (0.9%) did not identify themselves as being involved in culling decisions for their herd. About half (48.7%) of respondents answered they provide no special management practices for cull cows in the short term before a cow leaves the farm, and less than a quarter of respondents identified providing cull cows alternative housing (24.6%), a change in feed (20.1%), or milk dry-off practices (14.7%). About a quarter (21.9%; *n* = 49) of farmers identified they had received a letter from OMAFRA (sent when an animal is identified by a provincial inspector as being unfit for transport) about at least one cow they have had processed at a provincial sales yard.

Most respondents (69.9%) indicated that they were enrolled in milk recording programs with Lactanet (DHI) in Ontario Canada. The frequency of regularly scheduled veterinary visits (i.e., herd health visits) varied widely among respondents. The most common identified frequency was bi-weekly (44.7%). Other frequencies of veterinary visits from most often to least were weekly (4.9%), every 3 weeks (6.2%), monthly (23.9%), every 2 months (4.0%), less than every 2 months (1.8%), and having no regularly scheduled visits with a veterinarian (6.6%).

### Cull cow transportation

**Figure 1:**
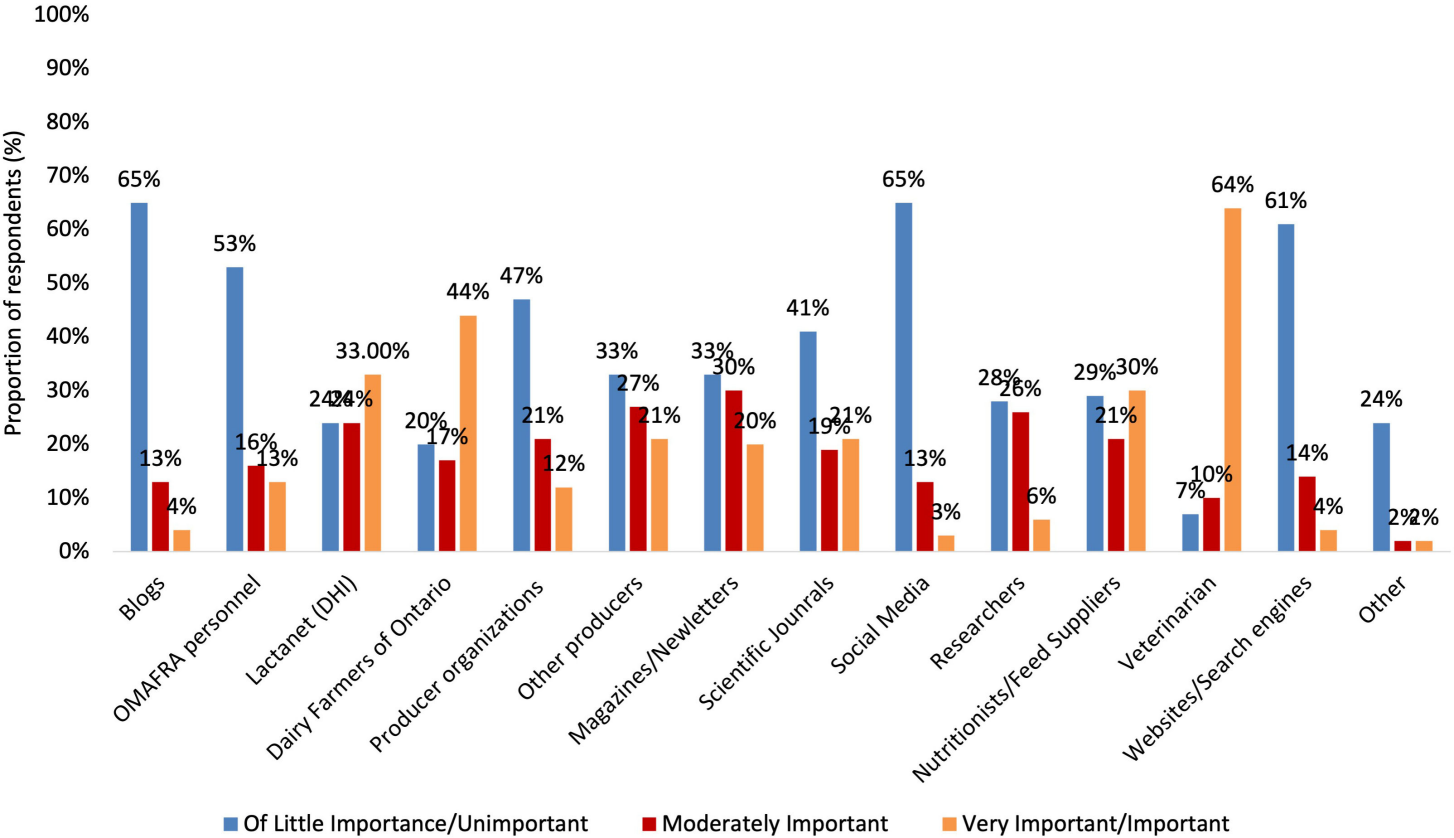
Proportion of respondents (*n* = 226) view on the importance (“very important/important,” “moderately important,” and “of little importance /unimportant”) of factors (body condition score, temperature, lameness, lactation status drug withdrawal time, mastitis, reproductive status, other disease/injury/illness, and ability of the cow to stand and stay standing for the duration of the trip) to assessment of cow fitness for transport immediately before loading, and whether or not factors were included in the farm cull cow standard operating procedure (*n* = 182).

Approximately three-quarters of respondents (74.6%; *n* = 167) identified that farm personnel re-assess the fitness of a cow for transport if there is a delay between deciding to cull a cow and when the cow leaves the farm. Most respondents identified the ability of a cow to stand during transport (73.2%; *n* = 156) as being very important to a cow's fitness for transportation and drug withdrawal status (69.6%; *n* = 164) as being very important to the allow ability of cows to be transported (Figure 1). The least likely factor to be considered very important to the assessment of a cow's fitness for transport was lactation status (5.8%) (Figure 1). Farmers identified the destination for the majority of cull cows (54.5%; *n* = 122) shipped from their farms is a sales barn. In addition to a sales barn, most respondents identified there were other destinations available, including directly to slaughter (73.2%; *n* = 164), a location determined by the transporter (17.4%; *n* = 39), or other (4.9%; *n* = 11). However, these destinations were the intended destination for less than a quarter of cull cows. Other destinations given by respondents were another farm, a local butcher, or a renderer for deadstock/salvage. Most respondents identified at least one on farm disposal method was available to them for cull cows including euthanasia by a veterinarian (58.5%; *n* = 131), euthanasia by farm personnel (37.1%; *n* = 83), and slaughter on farm (13.8%; *n* = 31). From leaving the dairy farm, most respondents (55.4%; *n* = 124) expected the journey for cull cows to slaughter was within 3 days. Only 17.8% (*n* = 40) of farmers expect their cull cows to spend more than 3 days off their farm before slaughter. Meanwhile, 8.5% (*n* = 19) of respondents were entirely unsure of the duration of time cull cows spend on the journey to slaughter.

### Cull cow standard operating procedures

**Figure 2:**
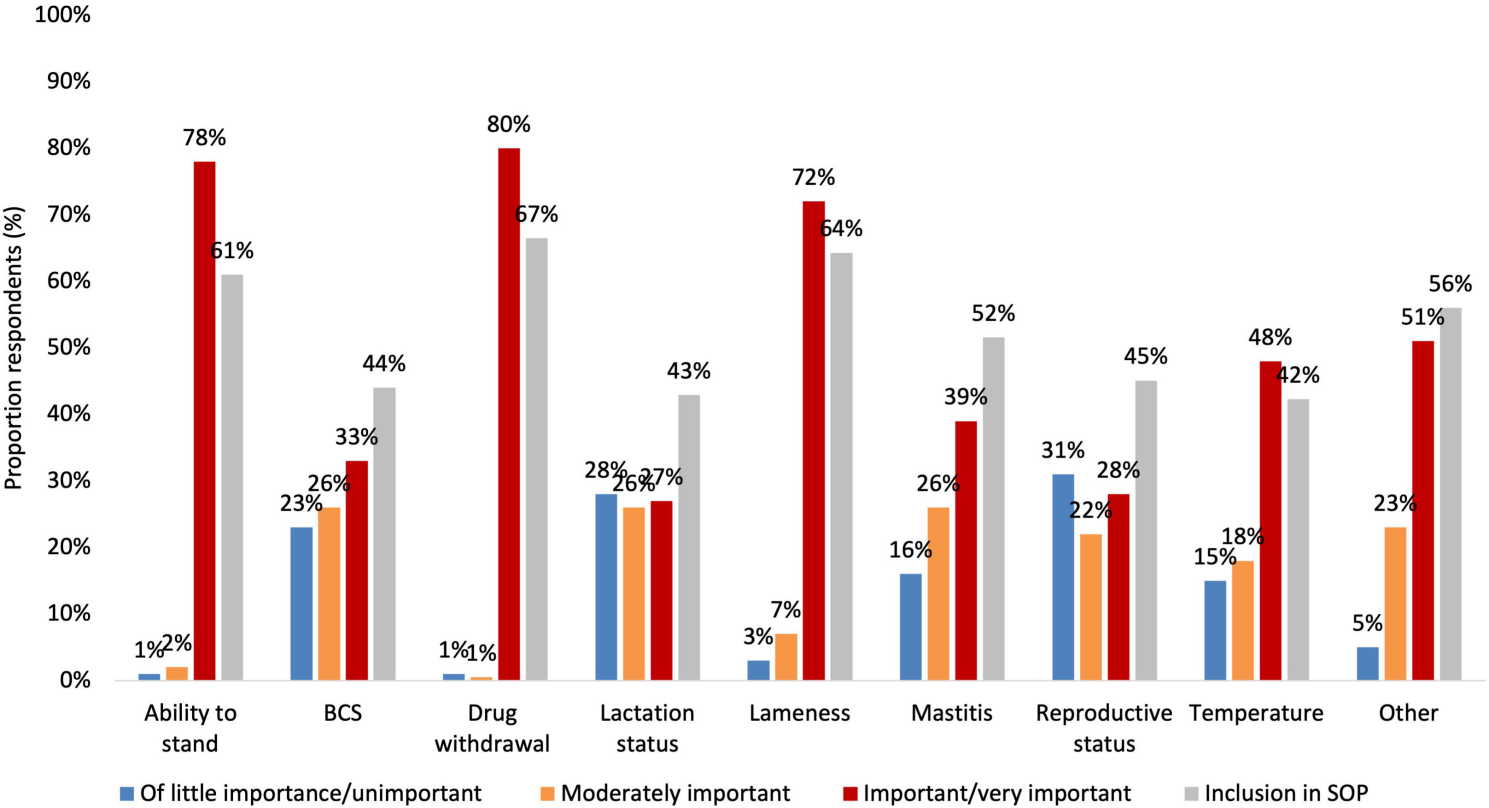
Proportion of respondents (*n* = 226) view on the importance (“very important/important,” moderately important,” “of little importance/unimportant”) of factors (blogs/online forums/LISTSERVS, extension personnel from OMAFRA, Lactanet (DHI), Dairy Farmers of Ontario (DFO), producer organizations other than DFO (e.g., OFA). Other producers, magazines/newsletters, scientific journals, social media (Facebook, Twitter, Instagram), researchers, your nutritionists or feed supplier, your veterinarian, websites/search engines (e.g., Google, Yahoo), and other) for obtaining information about cull cow management.

Most respondents (61.6%; *n* = 138) indicated that their farm has a written SOP for the management of cull cows prior to transport off farm. These SOPs were created with the assistance of a veterinarian almost half of the time (46.7%; *n* = 85). However, the minority (48.4%; *n* = 88) of respondents answered that they use this SOP every time a cow is being transported off farm, and 12.6% (*n* = 18) of respondents used their cull cow SOP more than a few times annually. Lastly, 14.8% (*n* = 27) of respondents identified never using their cull cow SOP. Over a third (36.3%; *n* = 63) of respondents identified updating their cull cow SOP annually, and similar proportions of respondents answered that they either update their cull cow SOP every 2 to 3 years (18.1%; *n* = 33) or when deemed necessary (20.9%; *n* = 38). The relative importance of various sources of information to assist with cull cow decision making is illustrated in Figure 2.

With respect to the logistic regression model evaluating whether farmers had a SOP for the shipping of cows, the farm role, age, education level, gender, cow housing system, frequency of veterinarian visits, enrolment in a milk recording program, role of culling decision maker, expectations of days to slaughter, and satisfaction with current knowledge of transportation regulations were offered to the multivariable model. The education level, cow housing system, frequency of veterinarian visits, and expectations of days to slaughter remained in the final model. Education level was not significant but was included due to a confounding relationship with the frequency of veterinarian visits. Multivariable regression identified that respondents who house their cows in tie-stalls were less likely to have a cull cow SOP than those farmers that house their cows in free-stall systems (OR = 0.24; *P* = 0.017) (Table 2). Respondents who more strongly believed there was not an impact on the condition of cull cows due to transportation were less likely to have a cull cow SOP (Table 2). Compared to other frequencies of veterinarian visits, those with bi-monthly veterinarian visits were more likely to have a cull cow SOP, and farmer education was seen to be a confounder of this relationship due to a difference of >20% in coefficients when it was removed from the model; therefore, it was kept in the final model.

**Table 2:** Final multivariable logistic regression model evaluating variables associated with the odds of complying with proAction requirements of having a cull cow standard operating procedure among 226 respondents.

**Variable**	**Count (Proportion)**	**Odds ratio**	**95% CI**	***P*-value**
**Education**				
Some public school	2 (0.9)	Referent		
Completed public school	7 (3.1)	0.34	0.007–15.5	0.581
Some high school/	46 (20.4)	0.11	0.004–2.59	0.170
Completed high school				
Apprenticeship training and trades	3 (1.3)	0.71	0.015–32.6	0.862
Completed college	90 (39.8)	0.53	0.027-10.4	0.676
Completed university/graduate School/professional degree	78 (34.5)	0.31	0.016–6.23	0.447
**Cow housing**				
Free stall	141 (62.4)	Referent		
Tie stall	57 (25.2)	0.28	0.096–0.792	0.017
Bedded pack	8 (3.5)	0.24	0.025–2.25	0.210
Other	1 (0.4)			
**Frequency veterinarian visits**				
Weekly	11 (4.9)	Referent		
Bi-weekly	101 (44.7)	4.11	0.469–36.0	0.202
Every 3 weeks	14 (6.2)	11.2	−0.927–136	0.057
Monthly	54 (23.9)	3.35	0.342–32.9	0.229
Bi-monthly/Less than every 2 month	13 (5.8)	27.4	2.04–368	0.012
No regular visits	17 (7.5)	6.06	0.468–78.4	0.168
Perception transportation impact^a^	224 (100)	0.83	0.686–0.993	0.042

a Predictor variable for farmers confidence level that cull cows who were removed from the respondent's farm arrived at slaughter facilities in the condition they were at the farm. Indicated confidence was on a scale of 0 (very low confidence) to 10 (very high confidence).

### Farmer confidence

Overall confidence of respondents that the cull cows removed from their farm over the last 12 months arrived at a slaughter facility in the same condition they left the farm was high. On a scale of 0 to 10 (with 10 being very confident), most respondents (58.0%; *n* = 105) indicated a confidence level of ≥8, and only 33 respondents (18.2%) rated their confidence as being <6 (Table 3). Multivariable analysis showed that farms that strongly believed transportation did not impact cow condition were less likely to have a cull cow SOP (OR = 0.83; *P* = 0.013; SOP (Table 2).

**Table 3:** The ranked importance of cull cow fitness assessment factors by that of information sources for cull cow management.

**Fitness assessment factor**	**Blogs/online forums/Listservs**	**OMAFRA**	**Lactanet (DHI)**	**DFO**	**Other producer organizations**	**Other producers**	**Magazines/newsletters**	**Scientific journals**	**Social media**	**Researchers**	**Herd nutritionist**	**Herd veterinarian**	**Websites/search engines**	**Other**
(1)^a^ Drug withdrawal time	7^b^ (3)^c^	27 (12)	71 (32)	27 (12)	27 (12)	46 (21)	44 (20)	45 (20)	6 (3)	58 (26)	66 (29)	142 (63)	8 (4)	8 (4)
(2) Ability of the cow to stand	7 (3)	25 (11)	70 (31)	25 (11)	25 (11)	44 (20)	41 (18)	44 (20)	**5**^d^ (2)	56 (25)	64 (29)	138 (62)	7 (3)	8 (4)
(3) Lameness	5 (2)	28 (13)	67 (30)	27 (12)	27 (12)	42 (19)	44 (20)	43 (19)	6 (3)	53 (24)	61 (27)	**129** (58)	7 (3)	5 (2)
(4) Other disease/injury/illness	6 (3)	**22** (10)	**50** (22)	21 (9)	21 (9)	32 (14)	34 (15)	34 (15)	5 (2)	43 (19)	47 (21)	92 (41)	6 (3)	5 (2)
(5) Temperature	5 (2)	21 (10)	45 (20)	17 (8)	17 (8)	24 (11)	27 (12)	32 (14)	6 (3)	40 (18)	44 (20)	90 (40)	5 (2)	5 (2)
(6) Mastitis	**6** (3)	**18** (8)	40 (18)	17 (8)	17 (8)	24 (11)	**31** (14)	29 (13)	6 (3)	32 (14)	**41** (18)	**75** (33)	4 (2)	6 (3)
(7) BCS	3 (1)	**15** (7)	33 (15)	12 (5)	12 (5)	18 (8)	19 (8)	23 (10)	4 (2)	28 (13)	29 (13)	63 (28)	2 (1)	5 (2)
(8) Reproductive status	2 (1)	**13** (6)	31 (14)	16 (7)	16 (7)	17 (8)	20 (9)	13 (6)	3 (3)	20 (9)	24 (11)	51 (23)	3 (1)	1 (1)
(9) Lactation status	3 (1)	13 (6)	23 (10)	11 (5)	11 (5)	19 (8)	18 (8)	16 (7)	4 (2)	23 (10)	26 (12)	49 (22)	2 (1)	5 (2)

a The ranked importance from most important to least important of cull cow fitness assessment factors for transportation.

b Count of respondents that ranked this fitness assessment factor and information sources as being important or very important to their assessment of cull cows' fitness for transport immediately before loading.

c Percentage of the 224 survey respondents that ranked this fitness assessment factor and information sources as being important or very important to their assessment of cull cows' fitness for transport immediately before loading.

d Numbers in bold represent a significant association (P < 0.05) between the cull cow fitness assessment factor for transportation and the information source for cull cow management.

### Information sources and importance of transportation assessment factors

Respondents assigned importance to each cow fitness assessment factor for transport had significant differences (*P* < 0.05) in their assigned importance to information sources for cull cow management. The importance farmers placed on different information sources varied by the components that they included in their SOPs. Relative to the factors included in SOPs, the most highly ranked information source by farmers was the herd veterinarian and the least highly ranked information sources were blogs/online forums/listservs and social media. All comparisons between information sources and fitness assessment factors for cull cows' management are presented in Table 3.

## Discussion

In comparison to the entire Ontario dairy farmer population, the study population had some notable differences including respondents being more educated and younger in age. Respondents were more likely to house cows in free-stall barns with parlor milking systems than the Ontario population of farmers, which largely houses cows in tie-stalls (28). It is possible that younger and more educated farmers were more interested in responding to the survey. However, the respondents' farm demographics still largely represent the target population of Ontario dairy farmers (Table 1).

Due to the financial importance of individual dairy cows to herd performance, it was expected most final decisions on when a cow is transported off farm were made by herd owners. Most research conducted investigating on farm management decisions have operated under the assumptions that decisions were being primarily made by single farmers, which has been the farm owner conventionally (29, 30). Quota management was the reason for culling the largest proportion of cows according to respondents, which may be a result of the unforeseen fluctuations in demand for some dairy products during the 2020 COVID-19 pandemic (31, 32). Reflecting previous research into culling decisions within quota management systems, 44.6% (*n* = 29) of respondents identified quota management as being responsible for at least a quarter of the total culling decisions made on their farm (33, 34). Another commonly cited reason for removal of cows is reproductive performance, which the Canadian Dairy Information Center reports being responsible for about 16% of culling decisions on Canadian dairy farms (5). The second most commonly cited reason for culling given by respondents was reproductive status, which 14.7% (33 of 224) respondents identified as being responsible for more than 25% of culling decisions on their farm.

In North America, most dairy cull cows are destined to pass through a livestock market on-route to slaughter (7). Thus, most farmers' expectation that a sales barn was the destination for more than 50% of the cull cows transported off their farm was an anticipated result. The second most identified destination for more than 50% of cull cows was a slaughter plant (i.e., direct slaughter; 28.1%), which numerous farmers commented they wish they had available for more of their cull cows as a destination. The lack of available local slaughter opportunities has been a regular topic for improved animal welfare within the Canadian dairy industry (14, 19). For at least some proportion of their cull cows, some respondents (20.1%; *n* = 45) were unaware of a cull cow's destination for transport, and this reflects an issue regarding the management of cull cows by farmers. Without the knowledge of the destination of a cull cow, farmers cannot always abide by rules and recommendations put in place, like proAction, to protect the welfare of dairy cows, especially animals considered compromised. The proAction initiative was created by the Dairy Farmers of Canada to improve animal health and welfare while ensuring milk quality and safety (2). If farmers are uncertain of the duration of travel of cull cows before slaughter, management decisions may lead to poor welfare outcomes for some cull cows (14, 35). This gap in knowledge may be at least partially responsible for findings of unfit cows at sales yards (16, 18, 19). A similar issue exists with farmers' expectations of the duration of time cull cows spend off farm before slaughter. In Canada, transportation prior to slaughter for cull cows may reach over 82 (+/−46) h and over 400 km from the farm (20, 36, 37). A study conducted in British Columbia reported nearly 10% of cows sold were slaughtered between 5 and 16 days after leaving the farm, and 2% of cows were observed for sale at two different auctions (36). In this study, most farmers expected their cull cows time to slaughter was within 3 days of leaving their farm. Therefore, the assessment by farmers of cull cows' ability to withstand the journey to slaughter may not be accurate.

Prior to transportation, farmers often assess a cow's ability to withstand the journey by reviewing different fitness related factors (14, 38, 39). More than a quarter (27.7%; *n* = 62) of respondents did not indicate that they assess a cow's fitness before loading onto a transportation vehicle. A similar number (28.1%; *n* = 63) identified lactation status as being either of little importance or unimportant to the assessment of a cow's fitness. However, drug withdrawal was identified as being either important or very important by most respondents (79.9%; *n* = 179) to the assessment of a cow before transportation, and although a majority, this proportion was concerning due to the importance of drug withdrawal to the legality of the shipment of cull cows. The importance assigned to drug withdrawal status in culling decisions was low compared to that previously found by Roche et al. (20), which reported 93% of Canadian dairy farmers identified drug withdrawal status as being important or very important. Prior to transport, Ontario dairy farmers are required to abide by drug withdrawal times, assess the capability of animals for transport, and milk lactating animals to avoid udder engorgement (22). Lactation status being a required part of assessing cattle fitness for transport is a relatively new expectation in comparison to drug withdrawal time, so this may have contributed to its lack of importance among some farmers (40). Several information sources were found to have a relationship to the level of importance farmers assigned to lactation status in assessing cow fitness for transportation, which suggests that farmers are attempting to familiarize themselves with why the factor has been added to regulations. The proportion of farmers (15.2%; *n* = 34) that considered mastitis as being unimportant or of little importance to the assessment of a cow's fitness before transport was concerning. Due to their causing weakness and discomfort, both a high lactation status and the presence of some levels of severity of mastitis represent factors of importance for animal welfare (14, 22). Most explicitly, this is because they likely will lead to a reduced ability to obtain feed and water and respond to external events like vehicle motion or other animals (14, 41). Similar to lactation status, BCS was considered either of little importance or unimportant to farmers 22.7% (*n* = 51) of the time. Moorman et al. (18) found cull cows with an unacceptable BCS (BCS ≤2) were significantly more likely to have an abnormal gait, and those with acceptable BCS (BCS >2) had higher price paid/kg than those without. Roche et al. (20) suggests body condition may directly contribute to a poorer state of welfare for cows under or overweight; however, the importance of body condition to the overall welfare of cows requires further research.

Respondents identified destinations and the proportion of cows sent to each destination that they have culled in the past 12 months. About a quarter (21.9%; *n* = 49) of farmers identified they had received a letter from OMAFRA about at least one cow they have had processed at a provincial sales yard. Although this study did not directly observe the condition of cull cows sent to slaughter, this reflects numerous recent findings demonstrating that some cull cows arrive at sale in conditions considered compromised or unfit with reduced welfare (16, 18, 39). Cattle are considered compromised when they are in peak lactation, have limited mobility, moderate to severe lameness, have a BCS of 2 or less, swollen or injured limbs, and/or signs of severe respiratory disease (2, 22). Additionally, according to the Canadian Food Inspection Agency (CFIA), unfit animals “are not to be transported unless [it is] to receive care recommended by a veterinarian,” and some common signs of an animal being unfit include being non-ambulatory, lameness causing pain or inability to walk on all legs, and being extremely thin. With only 61.6% (*n* = 148) of farmers identifying having a cull cow SOP and 48.4% (*n* = 88) of those farmers using this SOP every time they transport a cow off farm, it is possible this lack of scrutiny of cull cows may be responsible for some unfit animals being sent to auctions. This poor level of utilization of SOPs means farmers are not informing themselves on specific cull cow management requirements regularly. According to the survey in the current study, only 46.7% (*n* = 85) of respondents identified that a veterinarian was involved in creating cull cow SOPs. The lack of involvement of veterinarian in the development of cull cow SOPs and low regularity of updating SOPs with new research findings and regulatory changes, may contribute to the transport of unfit cows. The finding that farms housing cows in tie-stalls were less likely to have a cull cow SOP than those that house their cows in free-stall systems may be a result of older housing systems being related to older cull cow management practices, operation diversification (i.e., business size), or number of employees (30, 42). The finding that farmers were less likely to indicate having a SOP for shipping cows when they were more confident transportation to slaughter was un-impactful to cows may indicate that farmers are unaware of the possible time and impact of the journey to slaughter. Therefore, they may view a cull cow SOP as being unnecessary for the welfare of cull cows. However, this assumption requires further investigation.

Currently few special management practices are required for cull cows before transport, yet most dairy farmers (51.2%) are providing some form of special management for their cull cows (21, 22). This reflects industry sentiments (i.e., DFO) and veterinarian recommendations that management practices to optimize the well-being of all dairy cattle, including cull cows, be implemented on farms (2, 43). Additionally, farmers may be instituting management changes for cull cows to meet regulatory requirements for animals intended for the marketing system. However, the lack of specific recommendations for the care of cows earlier than immediately prior to transport represents a gap in knowledge within research and a potential area for improving the welfare of cull cows.

Overall, farmers ranked veterinarians (64%; *n* = 143), DFO staff (44%; *n* = 98), DHI staff (34%; *n* = 74), and nutrition company staff (30%; *n* = 68) as important or very important sources of information. Therefore, these groups are critically important for educating farmers on cull cow management. There were some interesting relationships identified between the ranking of information sources and the specific relative importance of some elements of cull cow fitness. For example, when OMAFRA was ranked as an information source of higher importance by respondents, they ranked BCS as being more important to cull cow's fitness. This may be a result of OMAFRA's support of assessing BCS for cull cows such as their recently published and distributed cull cow decision action card, which was first published and sent to every dairy farmer in the province by mail in 2019 (44, 45). Respondents relatively highly assigned importance to the DFO and herd veterinarians as sources of information may be related to the industry's proAction initiatives. The industry quality assurance program informs farmers of the importance of assessing cull cow fitness including lameness. The significant associations found between the importance of mastitis and several information sources may reflect the association of mastitis with numerous health and production factors for potential cull cows (46, 47). The multifaceted nature of mastitis may lead to farmers investigating its importance from numerous sources to solidify their opinion. The increasing publication of the importance of lactation status in cull cow decision making by industry and government organizations may be responsible for the association between higher ranked importance of magazines and newsletters and lactation status for culling decisions. Meaning, farmers have learned from these publications the importance of lactation status relative to the transport of cull cows. Finally, for those that ranked the ability of a cow to stand as important, the importance of social media and other sources for information regarding cull cows were lower. This may mean that those more scrutinous of social media's value as an information source use more reputable sources of information to determine the importance of factors for cull cow fitness for transportation.

### Survey limitations

Since this summary of cull cow practices in Ontario, Canada was derived from a survey, the responses given may not entirely represent the true practices on farms due to respondents curating their responses for social desirability, misunderstanding questions, being unable to answer questions accurately, and not responding to some questions. Possible sources of sampling bias include language and mode of survey circulation. The survey was only available in English, which may have led to some dairy farmers being excluded or misunderstanding questions due to language barriers, and invitations were sent to farms in magazines and emails (and were available online), which may not have reached some individuals or reached multiple individuals within an operation. In particular, these factors may have limited responses from Francophone dairy producers and Mennonite producers. Additionally, the lower than desired overall survey response supports the potential for non-response bias in this work. Further, the lower response rate may have also contributed to a reduction in the statistical power for identifying other potentially important but unidentified variables in the analyses. Respondents may also have had recall bias in answering some questions. For example, some primary reasons for culling may have been more easily remembered by respondents than others, and some terms, like direct to slaughter, may be interpreted with slightly different meanings by individuals. The data was collected between 2020 and 2021, and therefore, reasonably reflects practices and beliefs of farmers today in 2022. Recent regulatory changes may have yet to be fully considered for their impact on cull cow decision making by some farmers, which may explain why they were assigned with lower importance.

## Conclusions

This study described respondents reported current Ontario, Canada dairy farm management practices and perceptions of cull cow management. The results identified several gaps in farmer perceptions and actual cull cow management off-farm and demonstrates that the adoption of cull cow management requirements has substantial room for improvement. Educational tools, decision aids, and workshops could be developed for farmers to improve management decisions and increase regulatory compliance. Additionally, this will inform further research into on-farm cull cow management and attitudes toward regulatory and research recommendations for cull cows, all leading to improved cull cow welfare.

## Data availability statement

The datasets presented in this study can be found in online repositories. The names of the repository/repositories and accession number(s) can be found in the article/Supplementary material.

## Ethics statement

The studies involving human participants were reviewed and approved by Research Ethics Board University of Guelph. The patients/participants provided their written informed consent to participate in this study.

## Author contributions

TD secured funding for the study. TD and JM contributed to conception and design of the study. JM and LL organized the distribution of the survey. JM led the data management and performed statistical analysis, overseen by DH, DK, CM, SR, and TD. JM wrote the first draft of the manuscript. All authors contributed to the article and approved the submitted version.

## Funding

Financial support for this research was provided by the OMAFRA Agri-Food Alliance KTT program.

## Conflict of interest

Author SR was employed by ACER Consulting Ltd. The remaining authors declare that the research was conducted in the absence of any commercial or financial relationships that could be construed as a potential conflict of interest.

## Publisher's note

All claims expressed in this article are solely those of the authors and do not necessarily represent those of their affiliated organizations, or those of the publisher, the editors and the reviewers. Any product that may be evaluated in this article, or claim that may be made by its manufacturer, is not guaranteed or endorsed by the publisher.
